# Women’s experiences of disrespect and abuse in maternity care facilities in Benue State, Nigeria

**DOI:** 10.1186/s12884-018-1847-5

**Published:** 2018-06-07

**Authors:** Joy Orpin, Shuby Puthussery, Rosemary Davidson, Barbara Burden

**Affiliations:** 1 Institute for Health Research, University of Bedfordshire, Putteridge Bury, Hitchin Road, Luton, Bedfordshire, LU2 8LE UK; 2 School of Health Care Practice & Institute for Health Research, University of Bedfordshire, Putteridge Bury, Hitchin Road, Luton, Bedfordshire, LU2 8LE UK

**Keywords:** Disrespect and abuse, Maternity care, Health facilities, Nigeria

## Abstract

**Background:**

Disrespect and abuse (D&A) of women in health facilities continues to be a prevailing public health issue in many countries. Studies have reported significantly high prevalence of D&A among women during pregnancy and childbirth in Nigeria, but little is known about women’s perceptions and experiences of D&A during maternity care in the country. The aim of this study was to explore: 1) how women perceived their experiences of D&A during pregnancy, childbirth, and in the postnatal period in Benue State, Nigeria; and 2) how women viewed the impact of D&A on the future use of health facilities for maternity care.

**Method:**

Five focus group discussions with a sample of 32 women were conducted as part of a qualitative phenomenological study. All the women received maternity care in health facilities in Benue State, Nigeria and had experienced at least one incident of disrespect and abuse. Audio-recorded discussions were transcribed and analysed using a six-stage thematic analysis using NVivo11.

**Results:**

The participants perceived incidents such as being shouted at and the use of abusive language as a common practice. Women described these incidents as devaluing and dehumanising to their sense of dignity. Some women perceived that professionals did not intend to cause harm by such behaviours. Emerged themes included: (1) ‘normative’ practice; (2) dehumanisation of women; (3) 'no harm intended' and (4) intentions about the use of maternity services in future. The women highlighted the importance of accessing health facilities for safe childbirth and expressed that the experiences of D&A may not impact their intended use of health facilities. However, the accounts reflected their perceptions about the inherent lack of choice and an underlying sense of helplessness.

**Conclusion:**

Incidents of D&A that were perceived as commonplace carry substantial implications for the provision of respectful maternity care in Nigeria and other similar settings. As a country with one of the highest rates of maternal deaths, the findings point to the need for policy and practice to address the issue urgently through implementing preventive measures, including empowering women to reinforce their right to be treated with dignity and respect, and sensitising health care professionals.

## Background

Disrespect and abuse (D&A) of women in health facilities continues to be a prevailing public health issue in many countries that violates the human rights of women to be treated with respect and to be free from harm [1]. Disrespectful and abusive maternity care can be experienced in various forms such as being ignored, shouted at, and slapped by healthcare providers, and abandoned to deliver a child alone in health facilities [2–4]. Disrespectful and abusive care has broadly been categorised into seven domains as physical abuse, discrimination, non-consented care, non-dignified care, abandonment or neglect, non-confidential care, and detention in health facilities [5]. Globally, women tend to experience D&A during labour and childbirth at health facilities regardless of their socio-demographic characteristics such as age and level of education [5, 6]. Some groups such as adolescents, unmarried women, women with Human Immunodeficiency Virus / Acquired Immunodeficiency Syndrome and those from low socioeconomic status are, however, more vulnerable to experience D&A compared with their counterparts [5].

Numerous adverse consequences of D&A on women’s health and wellbeing have been reported including increased risk of birth complications [7]; poor self-rated health, sleeping problems, and signs of post-traumatic stress disorder [8]; and the reluctance to use health facilities [5]. Many factors have been identified to contribute to D&A in health facilities such as poor communication between women and healthcare providers; inadequate healthcare policies; prejudices from healthcare providers; and demoralisation of healthcare providers in poorly performing health facilities [2, 5, 9, 10].

Although there are variations in prevalence, reports of high prevalence rates of D&A among women during facility-based childbirth have been documented in several countries [8, 11, 12]. For example, approximately 13–28% women who accessed obstetrics and gynaecology services in Northern Europe experienced some form of abuse in healthcare centres [8]. Another multi-country study found that 1 in 5 women who attended antenatal care in maternity health facilities had at least one episode of abuse in six European countries – Sweden, Norway, Belgium, Estonia, Iceland and Denmark [11]. Considerably higher prevalence rates are recorded in some of the African countries [6, 13, 14] including Nigeria, with the prevalence rates among women during childbirth in health facilities ranging between 23.7–98% [15–17].

Qualitative studies have reported women’s subjective experiences of D&A during childbirth in some countries. A qualitative study, based on a Danish sample of postnatal women who had previously endured abuse, found that abuse in health care may have profound consequences on the reproductive lives of the women including adverse impact on sexuality, the desire to have children, and the expectations of the mode of delivery [18]. While describing their childbirth experiences, some women in Tanzania reported abusive events such as verbal abuse, discriminatory treatment, and being ignored [4]. Non-confrontational measures such as returning home and bypassing certain health facilities and/or healthcare providers, or accepting the mistreatment, were reported by women in response to the abuse [4, 19]. Four scenarios in health facilities - verbal abuse, slapping, physical restraint and refusing to help- were used to understand the acceptability of mistreatment among service providers and women in Guinea [19]. The findings showed that women did not always accept the scenarios of mistreatment except when the actions of the healthcare providers were perceived as intended to save the mother and child [19]. The service providers accepted mistreatment when they felt women were uncooperative, disobedient, or the lives of their unborn children were at risk during childbirth.

In Nigeria, very few qualitative studies have explored women’s experiences of disrespectful and abusive maternity care. Two of such studies have explored how mistreatment occurred and its acceptability among service users and providers [10, 20]. One study identified four scenarios of mistreatment in two health facilities in Abuja - verbal abuse, slapping, physical restraint and refusing to help a woman [20]. All the participants perceived the scenarios as acceptable and appropriate measures to make mothers comply with healthcare providers’ instructions for the safe birth of their child [20]. In the other study, both healthcare providers and women reported how they had either witnessed or experienced verbal and physical abuse and detainment at health facilities [10]. Beyond these studies, there has been little investigation into women’s experiences of D&A in Nigeria. This study, therefore, aimed to develop an in-depth understanding of women’s perceptions and experiences of D&A in maternity care facilities in Benue State, Nigeria. Based on the first phase of a qualitative study, this article specifically explores: 1) how women perceived their experiences of D&A during childbirth, antenatal and postpartum care; and 2) how women viewed the impact of D&A on future use of health facilities for maternity care.

## Methods

This paper presents findings from the first phase of a two-phase qualitative phenomenological study to understand women’s experiences of D&A in maternity care. Phenomenology as a qualitative approach enables the study of people’s ‘lived experiences’ of a social phenomenon [21, 22]. Its application in this study enabled the researchers to obtain an in-depth understanding of women’s experiences of D&A in maternity care. Interpretivism is the research paradigm adopted because it allowed the use of qualitative research methodology to understand the meaning of an individual’s subjective experience of a phenomenon [23, 24].

### Setting, participants and procedure for recruitment

This study was conducted in Makurdi, Benue State, Nigeria. Two health facilities - the Benue State University Teaching Hospital and the Epidemiological Unit, Benue State Ministry of Health - were selected because of their central location in Benue State’s capital and easy accessibility.

Participants were recruited using purposive sampling methods. In order to identify women who had experienced D&A during their maternity care, a screening tool was developed based on a review of the literature and the types of D&A outlined by previous studies [5]. The screening tool was used to determine if women have experienced at least one type of D&A or any other subjective experiences perceived as D&A during their maternity care. Women who reported as having experienced at least one incident of D&A on the screening tool were invited to participate if they were aged 18 years and above; had received antenatal, childbirth and postpartum care at a health facility in the last two years; and have had a normal vaginal birth. Women who have had caesarean sections or stillbirths were excluded.

After obtaining permission from the Head of Obstetrics & Gynaecology departments and the Chief Medical Directors, the first author (JO) approached the participants in person during their visit to the health facilities for the immunisation of their new-borns or for postnatal care. Potential participants were first briefed as a group while they were waiting for their appointments, and all the interested participants were taken to a separate room where detailed information was given about the purpose of the study, the eligibility criteria, and the ethical considerations. They were also given the participant information sheet and the screening tool described above. Eligible women willing to participate signed a consent form to confirm their approval. While providing consent, all the participants were given a brief questionnaire with a set of demographic questions including age, tribe, marital status, religion, level of education, employment status, and number of previous births. Among forty-eight women who were given the screening tool and participant information sheet, thirteen women did not meet the eligibility criteria, and three declined participation. Thirty-two women were finally recruited for the focus group discussions (FGDs).

### Focus group discussions

A total of five FGDs were conducted with 6–8 participants per group involving a total of thirty-two women. The discussions were moderated by the first author (JO) and were conducted in a room at the health facilities where privacy was assured. The discussions were audio-recorded with permission from participants and were later transcribed verbatim. The moderator also kept a diary during the period of data collection with field notes and reflections.

A topic guide was used along with prompts as necessary for conducting the FGDs. The topic guide was developed based on the objectives of the study. The participants were first prompted to describe their maternity care (antenatal care, childbirth, postnatal care) experience at the hospital; what they liked and disliked about the care received; and the interactions with healthcare providers. This was followed by questions on any perceived factors that may have influenced the care received and the effect of this on their future use of health facilities for maternity care. All the FGDs were conducted in September and October 2016.

### Data analysis

All the audio-recorded discussions were transcribed by the first author (JO). The second author (SP) reviewed the transcripts and the audio-recordings to confirm consistency and credibility of the data analysis process. The analysis was conducted based on a thematic approach (Braun and Clarke, 2006). All the transcripts were read repeatedly for familiarisation and to develop an in-depth understanding of the data. Using NVivo 11, themes relevant to the research objectives were identified, categorised and interpreted [25]. The application of an interpretive paradigm enabled the researchers to focus on understanding the meaning of women’s subjective experiences of D&A in maternity care [23, 24]. Extracts from the FGDs are used to illustrate the emerged themes. Numbers were used to denote participants to protect the participants’ identities and anonymity in the transcripts [26].

### Ethical considerations

Ethical approval was obtained from the Institute for Health Research Ethics Committee, University of Bedfordshire (IHREC681), Government of Benue State Nigeria (MOH/MED/261/Vol.II/707), and Benue State University Teaching Hospital (NHREC/08/11/2013B/2016/0020). Permission to access and recruit participants was obtained from the Head of Obstetrics and Gynaecology, Benue State University Teaching Hospital and the Chief Medical Director of the Epidemiological Unit, Benue State Ministry of Health. Verbal and written consent was obtained from all participants. Any identifying information was removed from transcripts prior to analysis to ensure anonymity.

## Results

The sample comprised of 32 women aged between 18 and 37 years. All the women were married; 93.8% (*n* = 30,) were Christians and 6.3% (*n* = 2) were Muslims; 84.4% (*n* = 27) had post-secondary education; 15.6% (*n* = 5) had secondary education; 65.6% (*n* = 21) were unemployed and 34.4% (*n* = 11) were employed. Nearly half of the women (*n* = 13) had one child; 37.5% (*n* = 12) had two children and 21.9% (*n* = 7) had three or more children. All the participants described at least one episode of D&A during their last maternity care in health facilities. The FGDs lasted between 40 and 75 min. The socio-demographic characteristics of each of the participants are presented in Table 1. Four main themes emerged in the women’s accounts: (1) ‘normative’ practice; (2) dehumanisation of women; (3) 'no harm intended'; and (4) intentions about the use of maternity services in future. The main and sub-themes are presented in Table 2.

**Table 1:** Socio-demographic characteristics of participants

Participants		Age range(years)	Tribe	Marital status	Religion	Level of education	Employment Status	Number of children
FGD1	P1	21–30	Igala	Married	Christianity	Post-secondary	Unemployed	1
	P2	21–30	Tiv	Married	Christianity	Post-secondary	Unemployed	1
	P3	21–30	Tiv	Married	Christianity	Post-secondary	Unemployed	2
	P4	21–30	Tiv	Married	Christianity	Post-secondary	Unemployed	1
	P5	21–30	Tiv	Married	Christianity	Post-secondary	Unemployed	1
	P6	21–30	Igede	Married	Christianity	Post-secondary	Unemployed	2
	P7	21–30	Igede	Married	Christianity	Post-secondary	Employed	3 or more
FGD2	P1	21–30	Idoma	Married	Christianity	Post-secondary	Unemployed	2
	P2	21–30	Idoma	Married	Christianity	Post-secondary	Unemployed	3 or more
	P3	21–30	Tiv	Married	Christianity	Post-secondary	Employed	2
	P4	20 and below	Tiv	Married	Christianity	Post-secondary	Employed	1
	P5	21–30	Tiv	Married	Christianity	Post-secondary	Employed	3 or more
	P6	21–30	Idoma	Married	Christianity	Post-secondary	Unemployed	2
	P7	21–30	Tiv	Married	Christianity	Post-secondary	Unemployed	1
FGD 3	P1	31–40	Tiv	Married	Christianity	Post-secondary	Unemployed	3 or more
	P2	21–30	Igede	Married	Muslim	Post-secondary	Employed	2
	P3	21–30	Tiv	Married	Christianity	Post-secondary	Unemployed	1
	P4	21–30	Tiv	Married	Christianity	Secondary	Unemployed	2
	P5	20 and below	Tiv	Married	Christianity	Secondary	Unemployed	1
	P6	21–30	Tiv	Married	Christianity	Post-secondary	Employed	2
FGD 4	P1	31–40	Tiv	Married	Christianity	Post-secondary	Unemployed	2
	P2	21–30	Igbo	Married	Christianity	Post-secondary	Employed	3 or more
	P3	21–30	Tiv	Married	Christianity	Post-secondary	Unemployed	1
	P4	31–40	Tiv	Married	Christianity	Secondary	Employed	2
	P5	21–30	Tiv	Married	Christianity	Post-secondary	Employed	1
	P6	21–30	Igbo	Married	Christianity	Post-secondary	Unemployed	1
FGD 5	P1	21–30	Idoma	Married	Christianity	Secondary	Employed	2
	P2	21–30	Tiv	Married	Christianity	Post-secondary	Unemployed	3 or more
	P3	21–30	Tiv	Married	Christianity	Post-secondary	Unemployed	1
FGD 5	P4	31–40	Igede	Married	Muslim	Post-secondary	Employed	3 or more
	P5	31–40	Idoma	Married	Christianity	Post-secondary	Employed	2
	P6	20 and below	Tiv	Married	Christianity	Secondary	Unemployed	1

**Table 2:** Main themes and sub-themes

Main themes	Sub-themes
‘Normative’ practice	Normal (mis)behaviour of healthcare providers
	Common practice in health facilities
	Endure abusive practices
Dehumanisation	Degrading women
	Lack of empathy
'No harm intended'	Encouragement to “push”
	Intended to save life
Intentions about the use of maternity services in future	No choice – helplessness
	Use different hospitals for childbirth
	Use private hospital instead of public hospitals

### ‘Normative’ practice

A key theme running throughout the accounts of the women was the systematic experience of verbal abuse from healthcare professionals, whereby such experiences became a normative practice for the women. Participants described how common it was to experience disrespectful and abusive practices themselves such as being shouted at with abusive language from healthcare staff or being treated harshly and to witness other women experience such incidents, especially during antenatal care and childbirth. Women quite often regarded such actions as a normal behaviour/misbehaviour from healthcare providers:

“*…the nurses at that antenatal [clinic] were not friendly at all; they were quite abusive... they use very harsh words and even be insulting women. Sometimes there is even no reason for the abusive language but it is a common thing at the hospital*” (FGD5, P2).

*“They treated me in a way that was not always good but that's how they always behave during our antenatal [appointments]. It's normal for them to behave like that anyway*” (FGD3, P3).

*“They shout at women... in fact; we are used to it”* (FGD3, P4).

One woman stated that abuse was a particular problem in government-funded hospitals:

“*For me, they were always too harsh, and this is common especially in government hospitals”* (FGD3, P2).

Three participants recounted how they coped with abusive practices with the hope that their time at the health facilities for antenatal care and childbirth would be over soon, and that they would return home:

*“When you ask them a question, they will just be shouting at you for nothing... but I just manage them till I finish antenatal [care] because I know I will soon go home*” (FGD 5, P4).

*“What she was saying plus the pains I started crying again and all I wanted is to endure everything so I can go home because that is how she was saying that to other women too*” (FGD3, P1).

Women appeared to endure such practices as they felt they were not allowed to express their needs or feelings and had no other choice:

*“They will not even give you chance to talk. They will just shout at you, but you have nothing to say or to do than to manage”* (FGD1, P1).

*“So, what you just have to do is that you will endure anything you see or anything that happens to you”* (FGD2, P6).

Some women described how they used their faith as a source of strength to cope with their experiences of disrespectful and abusive care at the health facilities:

*“I just endure and pray to God that it will not happen again when I go to the hospital”* (FGD5, P5).

“*So, some of them [health care professionals] can be very rude at times but it’s just God that will help us to really cope with them and manage how badly they treat us*” (FGD 2, P3).

### Dehumanisation of women

Dehumanisation, as reflected in the women’s accounts, referred to how they felt about losing their value and dignity while going through the experience of D&A during antenatal, childbirth and postpartum care. Some women reported that the experience of disrespectful and abusive actions destroyed their feelings of self-worth and made them feel like they were not treated as ‘human beings’. This was mostly reported by women who have had given birth to their first child:

*“The nurses there will be shouting at you as if we are not human beings”* (FGD1, P1).

“*It is bad. They make you feel like you are not human being. It's like they just like to shout and quarrel [with] pregnant women…*” (FGD2, P7).

*“They make you feel like you don't matter when they talk to women in a rude way and sometimes that can make a woman feel like she doesn't mean anything to them*” (FGD3, P6).

A number of participants described their experiences of D&A as a consequence of the healthcare staff having no empathy for women. Such responses were mostly from women who were cared for by female nurse-midwives during their childbirth. Participants had expected the female healthcare providers to recognize and empathise with the pain experienced during childbirth as fellow women who had undergone, or expected to undergo labour in the future:

*“They talk to women in a rude way, and it is bad because they are women like us, but they cannot even treat women well”* (FGD3, P6).

*“You know I was thinking they will at least be nice and try to understand, especially now that the baby was coming but they just didn't care. They were just angry and quarrelling [with] me*” (FGD4, P6).

### 'No harm intended'

Some women did not consider the behaviour of healthcare providers during labour and childbirth to be disrespectful and abusive. Instead, they viewed these behaviours as well intentioned. For example, actions such as being slapped or hit on their legs were considered as a reminder to exert the pressure required to safely give birth to their children. Women described how they sometimes had to close their legs during labour and childbirth because of the labour pains but when healthcare providers shouted, hit, or slapped them, they opened their legs again. For these women, the anger from health care professionals, or being slapped was a reminder of the need to ‘push’ or to exert the pressure necessary to give birth to a child. These women often reported such actions as intended to save the lives of their unborn children, specifically, during childbirth:

*“I like the care but as I was pushing, the nurse was shouting that I should stop disturbing her and when I did not push well, she slapped my leg and said that I will kill my baby if I close my leg and because of that, I open my legs. So, she help me to [give birth to] my child safely”* (FGD1, P5).

*“…when I was tired and not pushing, she was angry at me that I want to kill my baby and I was like trying hard to push. [The midwife] was okay, but when I don't push, she will get angry and quarrel [with] me to push”* (FGD3, P5).

*“At times we women we do close our legs. We know that that’s the only thing that will help us. Then they will shout at us, say open your legs madam, why are you closing your legs? Do you want to kill the baby? Then, you will come and open your legs*” (FGD1, P7).

Not all women, however, perceived being shouted at or slapped as a reminder to ‘push’ or save the lives of their unborn children. One woman recounted how her inability to ‘push’ made the healthcare provider angry and she had her childbirth induced as a result without her consent. She described feeling violated because she wanted to experience childbirth naturally, but the process was disrupted:

*“I was like it’s not my fault it’s when the baby wants to come out, but he said I was not pushing well, so he got angry and induced me like that without my permission. That was the annoying part!”* (FGD1, P3).

Another woman recounted an instance when she was slapped on the leg by a nurse for failure to ‘push’, but she considered it as an intentional act to cause her pain. She also perceived the intervention from another provider as a proof to support her view:

*“The other one was very harsh on me, that, I was like, I want to kill my baby, I don’t want to… she even slapped me at a point, but the doctor said she shouldn’t do that next time”* (FGD3, P3).

Some women, especially those who have had previous deliveries, felt the actions of healthcare providers were perpetrated without justification while reflecting on and contextualising their experiences:

*“Sometimes there is even no reason for the abusive language, but it is a common thing at the hospital*” (FGD5, P2).

“*They shout at you, for just no cause…*” (FGD2, P5).

### Intentions about the use of maternity services in future

The women’s views showed that they clearly appreciated the importance of using health facilities for their care during pregnancy and childbirth inorder to prevent complications and the loss of life during childbirth. Despite their experiences of D&A, participants had intentions of using and of encouraging other women to use health facilities for maternity care in the future because they perceived facility-based childbirth as safer than home births. However, a sense of helplessness from the lack of choice for a better health facility was apparent:

*“You don’t have [a better] hospital; you don’t have choice; there is nothing one can do*” *(FGD2, P6)*

While some women stated that they would return to the same health facilities for their next pregnancy, others intended to go elsewhere. Women who had experienced D&A during childbirth were mostly of the opinion that although they might come back to the same hospital for antenatal or other services, they would use a different facility for the care during childbirth as they felt particularly vulnerable during this time:

“*I’m not going to deliver there again; though I like their antenatal [care], but the delivery…I will not go there*” (FGD4, P5).

*“Where I go for antenatal [care], I don’t think I can deliver from that hospital and even this one too, as I was going there for antenatal [care], I change my mind I deliver somewhere else*” (FGD2 P2).

One woman stated that she would use government hospitals for antenatal care, but private hospitals for childbirth with the hope of receiving better services:

“*So, for delivery, I would choose to go to any other private hospital, no matter how they treat you, but I think their treatment will still be preferable to Federal Medical Centre”* (FGD2, P3).

## Discussion

This paper presented an exploration of women’s perceptions of their experiences of D&A and the perceived impact on their future use of maternity care facilities in Benue State, Nigeria. The women in this study reported several instances of D&A, but mostly in the form of verbal abuse at various stages of their maternity care – antenatal, childbirth and postpartum care. The findings suggest that D&A, especially in the form of verbal abuse, is a phenomenon commonly experienced by pregnant women using healthcare facilities in this study. Overall women perceived these incidents as devaluing and dehumanising to their sense of dignity. Nevertheless, it did not prevent their intended use of health facilities for maternity care because they felt they have no other choice for maternity care. Furthermore, they acknowledge the role of facility-based childbirth in reducing risk to themselves and their unborn child. While some women reported one or more episodes of D&A during their antenatal and/or childbirth, others experienced it during antenatal and postpartum care. However, only a few women reported any experience of D&A during postpartum care as it appeared that most of them did not receive or seek any form of postpartum care upon discharge from the health facilities. It may be that women were put off from seeking postpartum care because of their experiences, but this was not fully explored in this study. As reported by previous studies, the responses from women in our study suggested that the experience of D&A was more severe during antenatal care and childbirth at the health facilities [18].

Women perceived their experiences of D&A as dehumanising as they expressed feeling devalued and worthless from the actions perpetrated by healthcare providers during maternity care. This violates women’s right to be free from harm and maltreatment as highlighted in the Respectful Maternity Care Charter developed within the context of the human rights perspective [1]. Dehumanisation has been described as denying people of their human attributes; thereby, equating them to animals [27]. It is often characterised by humiliating and degrading behaviour or a lack of empathy and indifference towards people [27]. The perception of D&A as dehumanisation has also been reported women who were treated without respect and dignity in other studies. The lack of respect made participants feel vulnerable, as well as dehumanised and worthless [18].

Another common theme that emerged from women’s responses in our study is their perception of D&A, for example, the use of abusive language and shouting as ‘normal’ behaviours from healthcare providers in health facilities during maternity care; therefore, such practices were expected and not necessarily seen as a violation of human rights. Previous studies have also reported that women tended to view D&A as normalised behaviour in health facilities in some countries [4, 10, 13, 28]. For example, women in Tanzania often expected disrespectful and abusive care to occur because they regarded it as the standard of care [28]. The Tanzanian women in the study initially reported being satisfied with the care they received at the health facilities, but on probing further, they came up with detailed accounts of their experiences of D&A. [28]. Women who have become familiar with disrespectful and abusive care during childbirth were not likely to regard it as a crime against women’s rights to health [1, 5]. The normalisation of D&A among women may be the underlying reason for most of the women in our study to feel that they had to accept and endure disrespectful practices. Such notions of D&A could also be the reason for it to remain underreported and unchallenged.

Further, some women regarded D&A as actions from healthcare providers that were not intended to cause pain or harm. The intentionality behind D&A in maternity care has remained a controversial topic in the literature [10, 19, 29]. Freedman et al. (2014) have referred to D&A as any deviation from the acceptable standard of good quality care, conditions in the health facilities, and actions by healthcare providers that are intended to or perceived as humiliating women [28]. The perceptions of women about D&A as actions necessarily well- intended from professionals in order to save both their lives and their unborn children also highlighted the acceptability of D&A in maternity care among women in this setting. Another study from Nigeria has described how women who experienced physical abuse in the form of slapping and beating did not get upset because they considered it as a measure to ensure the safe birth of their children [10]. It has also been argued that D&A could be seen as a deliberate act from healthcare providers to exert control on their patients [29].

The women in our study reported that they would continue to use the existing health facilities in future for maternity care regardless of their experiences of D&A as they accrued considerable importance to receiving care. Contrary to our findings, other authors have reported that D&A in healthcare may limit the use of facility-based maternity care [18, 30, 31] as the experience could potentially reduce women’s confidence in the health facilities [5, 31]. Although D&A may not have deterred the use of health centres for participants in our study for antenatal care or during childbirth, they tended to view the future use of health facilities with a sense of helplessness to avoid complications or loss of life during childbirth, with some women reporting intentions of using other hospitals they felt may provide more respectful maternity care.

### Strengths and limitations

This is one of the few qualitative studies that have explored women’s experiences of D&A in maternity care in Nigeria, and the first such study to be conducted in the Benue state. The women who participated in the FGDs represented a range of socio-demographic characteristics and had attended both government and private health facilities for maternity care. The use of FGDs enabled participants to discuss their experiences of D&A in health facilities interactively with the participants stimulating each other to express their views. However, the use of FGDs may have prevented some women from fully disclosing their experiences within a group. In the next phase of this study, individual interviews will be conducted to explore women’s experiences in depth. The FGDs were carried out in the health facility premises. Although privacy was ensured and the researcher explained the independent nature of the research and her role, courtesy bias or the feeling they should give “positive” responses so as not to compromise their future care, may have influenced women’s responses. During recruitment, the first author (JO) addressed the women in a group; those who were willing to participate were screened and their eligibility assessed. The information sheet, screening tool and the consent form were provided in English as this is the official language in the region and the country. However, this may have led to the recruitment of women with better English language skills and a more formal education, and a possible exclusion of women with lesser education and poor communication skills. Another limitation is the use of qualitative research methodology, which, due to the relatively small sample sizes and the unrepresentative nature of sampling techniques, does not seek to generalise findings to the population as a whole. Therefore the findings may not be representative of all the women in the region or in Nigeria.

## Conclusion

Our study provided an understanding of how women perceived their experiences of D&A and its perceived impact on their use of maternity services providing valuable insights for maternity care policy and practice in the country and other similar settings. The normalisation of D&A among the women along with the feeling that they had to accept and endure practices that they found dehumanising, coupled with the perceptions of helplessness in their future use of maternity services are all key issues to consider towards tackling the issue. The evidence from our study points to the urgent need for sensitising women and other family members about right to respectful care and empowering them to report and challenge disrespectful and abusive practices. It also reflect the importance of sensitising and training healthcare providers on the importance of providing respectful care.

Large scale studies with a representative sample size are needed to determine whether women’s views of D&A reflected in this study can be generalised to the region and Nigeria as a whole as well as to other similar settings. Future qualitative studies should focus on examining the experiences of vulnerable women such as those with no formal education. Studies covering health care professionals’ views will also be of immense value towards developing a comprehensive understanding of the phenomenon.

## Abbreviations

D&A: Disrespect and Abuse
FGD: Focus Group Discussion
